# Untangling Trustworthiness and Uncertainty in Science: Implications for Science Education

**DOI:** 10.1007/s11191-022-00322-6

**Published:** 2022-02-04

**Authors:** Beth A. Covitt, Charles W. Anderson

**Affiliations:** 1 spectrUM Discovery Area, University of Montana, Missoula, MT USA; 2 Department of Teacher Education, College of Education, Michigan State University, East Lansing, MI USA

## Abstract

This article focuses on *uncertainty—*ways in which scientists recognize and analyze limits in their studies and conclusions. We distinguish uncertainty from (un)trustworthiness—ways in which scientific reports can be affected by conscious deception or unconscious bias. Scientific journal articles typically include analyses and quantifications of uncertainty in both quantitative forms (e.g., error bars, ranges of predictions, statistical tests) and qualitative forms (e.g., alternate hypotheses, limitations of studies, questions for future research). These analyses of uncertainty are often incorporated into reports from scientific organizations and responsible scientific journalism. We argue that a critical goal of science education should be to help students understand *how* science may be employed as an uncertain and limited, yet still useful tool for informing decisions about socioscientific problems. When members of the public are insufficiently prepared to understand analyses and quantifications of uncertainty, the consequences are manifest in public skepticism about science and inadequately informed decision-making about socioscientific issues. We describe current design work in science education that includes a worthwhile emphasis on helping students to recognize and leverage uncertainty in their own data and models. Additional important work can enable students to develop proficiency in seeking out and understanding analyses of continuing uncertainty in media accounts of scientific conclusions and predictions.

## Introduction: Trustworthiness and Uncertainty in Science

Public trust in science in what has been characterized as our post truth world is a critical problem facing science education and a problem with no simple solutions (Barzilai & Chinn, 2020; Chinn et al., 2020; Feinstein & Waddington, 2020; Kienhues et al., 2020; Oreskes, 2019). In this article, we shift focus from the question of “why” people should trust science to that of “how” people should trust science. We argue that there are several distinct issues that require attention as people figure out how to evaluate science and the outputs of the scientific enterprise. One issue concerns the *trustworthiness* of science, another concerns *uncertainty* stemming from inherent aleatory and investigatory limitations of the scientific enterprise.

Both issues are important and relevant to the problem of public (dis)trust in science, yet these concerns are frequently conflated in the science education literature and science education instruction. Our article tilts focus toward the issue of uncertainty not because we think uncertainty is more important than trustworthiness, but because we believe that scientific analyses of uncertainty deserve increased attention in science education. We begin with a statement of our perspective of science followed by a discussion of the relationship between trustworthiness and uncertainty in the scientific enterprise. While acknowledging that there are many views and definitions of science, here we adopt the perspective that science is an international subculture (or group of communities of practice) that shares a social language, established channels of communication (including journals), and common knowledge and practices.

The issue of *trustworthiness* of scientific claims and predictions is evident in the call for this special issue, which draws our attention to “claims that science suffers from a systematic bias through sexism, racism, capitalism, colonialism and other ideological interests.” Cultural critics of science argue that scientific conclusions are not trustworthy due to bias, unexamined assumptions, self-interest, and other sources of human-fallibility (e.g., Bang et al., 2012, 2018; Benjamin, 2013; Gunckel, 2022; Jamieson, 1996). These critics appropriately exhort scientists to acknowledge and redress culturally biased assumptions, beliefs, and practices.

**Fig. 1:**
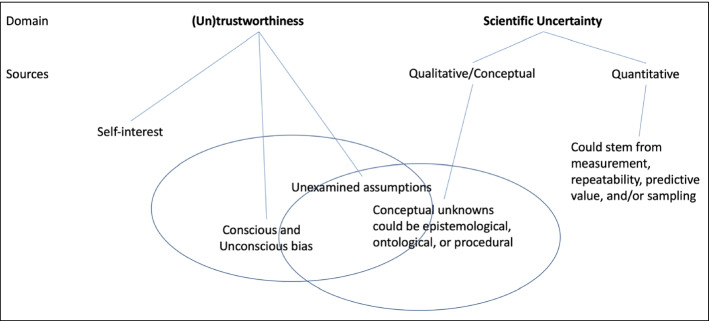
Perspectives of (un)trustworthiness and uncertainty in science (modified from Kirch, 2012)

While external critics of science often focus on trustworthiness, communications within scientific communities often focus on scientific uncertainty. Figure 1 helps illuminate the distinctions as well as the areas of overlap between trustworthiness and uncertainty. For example, Fig. 1 represents uncertainty as having quantitative and qualitative sources. The domain of scientific uncertainty that tends to be most distinct from trustworthiness concerns quantitative analyses of uncertainty. Both scientists and external critics of science commonly identify qualitative aspects of scientific uncertainty. However, this area of overlap might be characterized differently by the two communities; whereas critics of science might call attention to unexamined assumptions as reflecting conscious or unconscious biases, scientists might describe qualitative aspects of uncertainty as alternate hypotheses or unanswered questions.

Concerns about trustworthiness and uncertainty are not unique to science. People in all cultures and communities of practice have developed language and practices for dealing with trustworthiness and uncertainty, and there is no one community whose approaches are always superior. These issues are particularly important in education, where we must decide “whose certainty” or “whose uncertainty” should be taught, and for socioscientific issues, where we must make personal and collective choices about courses of actions based on uncertain information.

We argue that science education should enable students to enter a dialogue that attends to both social critiques of science as inherently untrustworthy and scientific analyses of how research findings are inherently limited by uncertainty—yet still worthwhile and useful. We believe that an understanding of science and scientific findings as uncertain, limited, *and* useful is particularly critical for science education at this juncture in time, when humanity is faced with extraordinary threats such as climate change, degradation of water quality and availability, and global pandemics. This is because while science is limited and uncertain (and sometimes, yes, untrustworthy), it is also a uniquely important tool for addressing socioscientific problems.

We will also argue that a critical goal of science education should be to teach students *how* science may be employed as an uncertain and limited, yet still useful tool for informing decisions about socioscientific problems. In making this point, we note that endorsing consideration of scientific conclusions and predictions within discussions and debates is not the same as endorsing policies or actions based on those conclusions and predictions. Political leaders who claim that their policies are “following the science” are never merely following the science. They are using scientific conclusions and predictions as support for policies that are also based on many other political, ethical, economic, and social considerations (Feinstein & Waddington, 2020).

This article focuses mostly on how science education could help students understand scientific analyses of uncertainty and use their understanding to inform (but not determine) their opinions and actions related to socioscientific issues. In several places, we discuss ways that instruction addressing scientific analyses of uncertainty can intersect with the issue of the trustworthiness of science. To make our case that science education should offer instructional experiences that engage students in learning how science is limited, uncertain, and useful, we will consider four issues.

First, making sense of scientific uncertainty requires some understanding of how the scientific enterprise works—particularly with respect to how scientists make claims about uncertainty in both settled and cutting-edge science. Thus, in Sect. 2, we discuss how claims about uncertainty are communicated among scientists, both in reports of settled science, such as science textbooks, and reports of cutting-edge science, such as scientific articles.

Because our argument is concerned with the interface between communities of scientists and the public domain, in Sect. 3, we consider scientific claims about uncertainty from perspectives outside of the subculture of Western science. This discussion includes two parts. One focuses on evidence, often from psychology and communications fields, concerning public awareness and understanding of scientific claims about uncertainty, including differences between scientific and lay ways of perceiving uncertainty within scientific discourse. In addition, we discuss perspectives on scientific claims about uncertainty and approaches to risk and uncertainty found in other cultures. We suggest that for both scientists and the public, there is a tension between two ways of responding to claims about uncertainty: *epistemic hubris* rejects claims about uncertainty and asserts the superiority of favored claims; alternatively claims of uncertainty can invoke *curiosity and information seeking*.

Next, Sect. 4 discusses different ways that scientists and journalists portray scientific claims about uncertainty in public-facing communications. We focus on communications about cutting-edge science and discuss approaches including omission of uncertainty, portrayal of uncertainty as a source of contention or disagreement, and portrayal of claims about scientific uncertainty as an expected and normal part of the scientific endeavor.

Finally, in Sect. 5, we address implications for science education. Recent science education literature documents an increasing focus on uncertainty (e.g., Chen et al., 2019; Kirch, 2012; Manz & Suárez, 2018; Metz, 2004; Pallant et al., 2020; Schroeder et al., 2019). Much of this work foregrounds instructional approaches that encourage students to respond to uncertainty with curiosity and information seeking. There is less research focused on instruction that helps students make sense of analyses of continuing uncertainty that cannot be readily resolved as part of a pedagogical sequence (e.g., Chen et al., 2019). We note that conceptual (qualitative) uncertainty has received more attention in education and may also be more easily accessible for students to make sense of compared with quantitative or statistical analyses of scientific uncertainty (Mayes et al., 2014). Thus, we argue that helping students learn to generate and make sense of scientific quantitative analyses of uncertainty deserves attention and point to promising initiatives of two types:

1. Approaches to engaging students in analyzing and quantifying uncertainty in data collection and in their analyses of data and models
2. Approaches to helping students evaluate scientific claims about uncertainty in media reports of scientific conclusions and predictions as a critical science practice that can be applied to inform decision-making about socioscientific issues

## Scientific Claims About Uncertainty in Cutting-Edge and Settled Science

We begin with a brief review of studies of the rhetoric of science in scientific primary literature—especially journal articles. We see that quantitative and qualitative analyses of uncertainty are pervasive in scientific primary literature, including both uncertainty that authors claim to resolve through their data collection and analyses, and continuing uncertainty that remains to be addressed through future research (e.g., Bazerman, 1988; Latour & Woolgar, 1979; Strevens, 2011). We also consider how *cutting-edge science*, as exemplified in the form and function of scientific journal articles, evolves into *settled science* as it is represented in science textbooks—what Fleck (1935/2012) referred to as “genesis and development of a scientific fact.”

### Claims About Uncertainty in Cutting-Edge Science

Although popular images of science can describe scientists as discovering indisputable facts, communication among scientists in journal articles is epistemologically far more complex. Students of the rhetoric of science (e.g., Bazerman, Fleck, Latour and Woolgar) point to ways in which scientific journal articles are organized as arguments in support of warranted conclusions. The stated conclusions, though, occupy only a small portion of a typical article; much of an article is devoted to discussions of uncertainty in various forms (Guillaume et al., 2017).

*Describing and reducing uncertainty:* A journal article typically begins with a literature review that describes uncertainty in the field—continuing issues that have not been fully resolved. This leads to research questions or hypotheses—statements of purpose about how the study will address these uncertainties. The methods section describes strategies for managing and reducing uncertainty through data collection and analysis. The results and discussion sections include claims about new knowledge that reduces the uncertainty described in the literature review.

*Claims about continuing uncertainty:* Scientific journal articles rarely describe conclusions as entirely certain. Instead, they include claims about continuing uncertainty that are reported, analyzed, and quantified in several ways, including, first, strategies for reporting and quantifying uncertainty in results: error bars for measurements, scatter plots, discussions of possible sampling bias, statistical analyses of the strength of patterns in data, etc.; and second, explicit acknowledgement of study limitations: remaining sources of uncertainty that could not be resolved. Finally, there are implications for future research: suggestions for additional studies that could resolve some of the continuing uncertainty from the current study (Guillaume et al., 2017). These claims about uncertainty can encourage a stance of continued information seeking—identifying what we don’t know as a spur to future research.

### Comparing Uncertainty in Cutting-Edge Science and Settled Science

Latour and Woolgar (1979) describe the nuanced ways in which scientists communicate about the degree of uncertainty in scientific claims, in which conjectures or hypotheses, when they are supported by data and analyses, become findings that are used by other scientists, with citations so that readers can establish their provenance and veracity. They further describe how some findings become so widely used that they are accepted within the field as *facts*, no longer needing citations to support them. For instance, physicists writing about the effects of forces on motions of objects do not need to cite Newton to justify that *f* = ma when they invoke that relationship in an argument or explanation.

*Textbooks as settled science:* The contents of high school and undergraduate college textbooks often consist almost entirely of settled science, that is, conclusions that are so widely accepted in scientific communities that they no longer need qualification or justification (Abd-El-Khalick, 2002; Chiappetta & Fillman, 2007; Knain, 2001). Thus, the textbooks follow the norms of scientific communities in presenting their conclusions without qualification. However, the rhetorical effect of this focus on settled science is significant for students. Claims about continuing uncertainty and signals about degrees of uncertainty that are found in the primary literature are culled out, leaving a rhetoric of conclusions that leads to an image of science as settled and certain. This image is problematic given that most of the science that can inform decisions concerning pressing socioscientific issues is unsettled and characterized by significant continuing uncertainty—more like journal articles than textbooks.

### Cutting-Edge Science in Socioscientific Issues

The continuing qualitative and quantitative uncertainty characteristic of cutting-edge science has important implications for how we understand and respond to socioscientific issues such as climate change and viral pandemics. The threats posed by such issues necessitate timely responses in the form of societal decisions and actions. However, understanding of these issues and associated predictions, for example, about what the climate will be like in 50 years or how high mortality rates for COVID-19 will be, is associated with significant uncertainties. Thus, societal decisions and actions concerning these issues must be taken in the context of both distrust of science and scientific uncertainty (Lee, 2012).

The community of science and related fields such as risk analysis have developed approaches to making predictions based on uncertain data, patterns, and models. For example, probabilistic approaches to making predictions include Bayesian inference (Ellison, 1996, 2004; Silver, 2012) and risk assessment (Eduljee, 2000; Gerba, 2006). These approaches to analyzing and quantifying risk are deeply embedded in fields such as engineering, finance, and public health, wherein quantified risk plays a critical role in decisions about design, allocating resources, and planning for contingencies.

## Perspectives on Uncertainty in Different Communities of Practice

In Sect. 2, we highlighted how specialists, working in data-rich environments, use conceptual and statistical tools to analyze and communicate uncertainty. In this section, we compare specialists’ approaches to those of two other communities of practice. First, we consider members of the public, who must make judgments about uncertainty and risk with limited time and data. Second, we consider Indigenous communities, who may have deep experience with uncertainty and risk spanning many generations in the places where they live, but whose knowledge is encoded in language and practices unfamiliar to Western scientists. We consider two possible outcomes of encounters between specialists and other communities. The first is *epistemic hubris*, wherein members of one or both communities harden their positions and discredit alternate approaches to uncertainty and risk. The second is *information seeking*, wherein uncertainty engenders curiosity and a desire for more information.

### Differences Between Lay and Specialist Perspectives on Uncertainty And Risk

Extensive scholarship has examined how people perceive and make sense of uncertainty (e.g., Broomell & Kane, 2017; Budescu et al., 2012; Dieckmann et al., 2017). This work dates to some seminal pieces such as Tversky and Kahneman’s (1974) “Judgment Under Uncertainty: Heuristics and Biases” and Slovic’s (1987) “Perception of Risk.” Many of these studies are located within the discipline of psychology. They point to differences between how lay people and specialists understand and perceive uncertainty, including scientific claims about uncertainty.

One prevalent finding is that lay people often pay attention to and care about different aspects of uncertainty compared with scientists and other specialists such as risk assessors. Whereas scientists and risk assessors focus on technical and quantitative aspects of uncertainty (e.g., probabilities or average measurements with error bars), lay people often focus on qualitative issues such as credibility or on characteristics such as risk knowability or dread (Slovic, 1987). Early on, these differences were conceived of as deficiencies in public understanding that science and risk communication specialists needed to fix by better conveying technical and quantitative information, so that the public could make decisions about uncertainties and risks using the same understanding as that held by specialists (Frewer, 2004; Hilgartner, 1990). This perspective has been labeled a “deficit model” of public understanding of uncertainty (Frewer, 2004). Over the past several decades, risk scholarship and practices have undergone a “cultural shift from more top-down communications to more consultative, transparent, and inclusive decision-making processes” (Frewer, 2004, p. 392).

Risk psychologists like Paul Slovic (1987) helped us understand that peoples’ responses to and decisions related to uncertainty and risk might be less related to quantified probabilities of harm and more related to perceptions of things like voluntariness, dread, knowability, and controllability. Nuclear power provides a classic example of this discrepancy. Because risks associated with nuclear reactors “are perceived as unknown and potentially catastrophic” (p. 285), they are viewed as more serious by the public than by nuclear industry experts. Like those who have pointed out the problems with a deficit model of public understanding, Slovic suggests that public perceptions of uncertainty and risk are valid, even though they are different from the perceptions of specialists. He argues that, “[l]ay people sometimes lack certain information about hazards. However, their basic conceptualization of risk is much richer than that of the experts and reflects legitimate concerns that are typically omitted from expert risk assessments” (Slovic, 1987, p. 285). Slovic and others warn against what Scott (2008) describes as *scientism*: unwarranted belief that scientific methods and conclusions always lead to the best course of action. Informed citizens understand that, like other scientific claims, scientific claims about uncertainty and risk are themselves uncertain, in ways that scientists themselves do not fully understand.

While lay perceptions of risk and uncertainty have value, scholarship concerning public understanding has also uncovered multiple challenges that people encounter when reasoning about scientific analyses of uncertainty. Some of these difficulties include relying on cognitive heuristics to make quick and snappy judgments when making careful inferences from scientific analyses is possible and would be useful (Covitt et al., 2021; Kahneman, 2011); perceiving expressions of scientific uncertainty as evidence of lower value, credibility, and usefulness (Broomell & Kane, 2017; Flemming et al., 2015; Rabinovich & Morton, 2012); and confusing competing scientific hypotheses that have the potential to be resolved through further investigation with political, economic, or other socially rooted disagreements that could not be resolved through science (Dieckmann et al., 2017; Rice et al., 2018).

### How Epistemic Hubris Can Thwart Meaningful Consideration of Scientific Uncertainty

An important socially mediated barrier to productive consideration of uncertainty is *epistemic hubris* or “the expression of unwarranted factual certitude” (Barker et al., 2021, p. 1). Epistemic hubris is not limited to any one community; scientists and non-scientists alike are prone to this type of thinking. While processes such as peer review and norms for analyzing uncertainty may provide some guardrails against epistemic hubris in communications within scientific communities (e.g., Guillaume et al., 2017); those guardrails often come down in communications between scientific communities and other communities. For example, scientific communications aimed toward public audiences often omit or oversimplify analyses of uncertainty that are present in insider communications (Ruhrmann et al., 2015; Stocking, 1999). The resulting communications portray scientific claims with more certitude than is justified.

In contrast, communications and expressed concerns about risk and uncertainty that come from “outsiders” are given less credence by Western scientists, especially if those communications are not conveyed using the conventions of scientific social language (Snively & Corsiglia, 2001). In these instances, epistemic hubris can lead Western scientists to conclude that inputs from other sources are not needed and information seeking in the investigatory process is prematurely concluded (Bang et al., 2018).

An example of reaching conclusions prematurely is evident in the Environmental Protection Agency’s (EPA’s) response to the Gold King Mine Spill (Beamer et al., 2016; Gunckel, 2022). In 2015, EPA personnel and other workers accidentally breached a mine tailings dam and released about three million gallons of acid mine drainage into a tributary of the Animas and San Juan Rivers. Risk assessment conducted by the EPA focused on impacts on recreational users and concluded that the spill did not pose a significant health risk (EPA, 2015; Gunckel, 2022). Gunckel further describes the case as follows:

However, the EPA did not take into account all of the ways that Indigenous communities along the river used the water, including for farming, drinking, and spiritual practices (Chief, 2016). From the perspective of the Navajo farmers affected by the spill, the limited assumptions about who used the water and for what purposes undermined the validity of the scientific model for making decisions about whether to use the river water to irrigate their corn. In contrast, a more culturally inclusive study was conducted by Chief et al. (2017) … [T]he researchers identified over 40 ways in which the Navajo community members used the water from the San Jan River. The researchers then investigated how lead and arsenic from the spill moved along the pathways that had the greatest potential to impact community members.

Further, in contrast with the EPA’s conclusion, a later study undertaken by Diné scientists found ongoing detrimental impacts of the spill on the Diné people—encompassing activities associated with diet, livelihood, recreation, and culture (Van Horne et al., 2021).

The literature documents many examples of Western scientific hubris, such as “scientific forestry” in the nineteenth century (Scott, 2008) and the origins of the Irish potato famine (Fraser, 2003a, 2003b). Many of these examples involve scientists’ incomplete understanding of complex systems or failure to consider traditional ecological knowledge (Snively & Corsiglia, 2000). Epistemic hubris can limit Western scientists’ curiosity and information seeking, leading to failure to consider important sources of uncertainty. Thus, integration of multiple perspectives, including perspectives from outside the culture of Western science, has the potential to meaningfully improve the quality of scientific conclusions, including analyses of risk and uncertainty.

While it can be difficult to judge whose perspectives should have standing in scientific investigations and arguments, one heuristic approach to evaluating such merit is depth of experience. In cases where, in retrospect, we conclude that critics of Western science were correct and Western scientists were wrong, the critics often had deep and relevant experience with the systems and phenomena being considered—experiences that scientists dismissed as unscientific traditional practice because it was not encoded in Western science language and practices (Bang et al., 2018; Snively & Corsiglia, 2000). Epistemic hubris and the case of the Gold King Mine spill reinforce the important idea that no one community or culture can stake a claim of possessing the best approaches to examining uncertainty.

## Public-Facing Communications Addressing Scientific Claims About Uncertainty

After people who do not become scientists finish school (where they are generally exposed to a rhetoric of science as certain and settled facts), they continue to encounter science communications in their lives. These communications come in a multitude of forms and have various ways of addressing scientific uncertainty. Some communications are designed to convey settled science in interesting ways, for example, through media such as nature films and museum exhibits. These communications often adopt the settled and certain rhetoric of conclusions like that found in schools.

Other communications, however, are intended to convey information about socioscientific issues, which generally involve claims from cutting-edge science that are subject to some combination of qualitative/conceptual and quantitative uncertainty. An example in which qualitative/conceptual uncertainty is more prominent comes from an April 2021 *Atlantic* article that discusses four competing theories for what mechanism underlies the rare blood clotting problem that has been associated with receiving the AstraZeneca and Johnson & Johnson COVID-19 vaccines (Khamsi, 2021). This article emphasizes the qualitative uncertainty surrounding a conceptual unknown—i.e., which of these different mechanisms causes blood clotting? An example in which quantitative uncertainty is more prominent comes from a February 2021 *New York Times* article that discusses conflicting advice concerning whether pregnant women should be vaccinated for COVID-19 (Mandavilli & Rabin, 2021). The source of uncertainty in this article stemmed from the fact that pregnant women were not initially included in vaccine trials, resulting in a lack of a sufficient data needed to evaluate the safety and effectiveness of COVID-19 vaccination for this group with standard statistical tests used in medicine. Though some articles may emphasize qualitative aspects of scientific uncertainty while others emphasize quantitative aspects, both facets of uncertainty are always present.

Note that in this article we focus on scientists and journalists producing legitimately intentioned communications with the aim of providing scientific information. Today, there are also many illegitimately intentioned communications that have aims such as disinformation and malinformation (Wardle & Derakhshan, 2018). Other scholars have addressed approaches for helping members of the public evaluate ill-intentioned science communications (e.g., Sinatra & Lombardi, 2020; Wineburg & McGrew, 2019), an important topic that is mostly outside the scope of our article. We draw a distinction between disinformation and malinformation (which are intentionally designed to deceive or mislead) and legitimately intentioned communications. We acknowledge that legitimately intentioned communications still inevitably reflect the biases and underlying aims of the communicator (Ebeling, 2008; Jensen, 2008; Kimmerle et al., 2015). There is no such thing as a completely objective portrayal of science in the media. While acknowledging the inevitability of bias, within the domain of legitimately intentioned communications about unsettled science written for public audiences, we observe several approaches to conveying scientific uncertainty.

### Conveying Unsettled Science as Settled and Certain

Sometimes scientists and journalists rely on the rhetoric of conclusions that is reflective of settled science. This type of communication misrepresents the discourse from the primary literature, winnowing out the scientific language of uncertainty and conveying findings and conclusions as settled (Ruhrmann et al., 2015; Stocking, 1999).

Rennie (2020) noted this type of communication approach in scientists presenting a series of publicly attended museum lectures concerning human genetics. She conjectured that the scientists simplified their findings to make a complex topic understandable to the public, consequently leading the audience to assume that the science content was more certain than was actually the case. This rhetorical move by scientists and journalists may stem from a belief that members of the public have poor understanding of scientific uncertainty and/or expect that science communications will convey science as certain (Cordner & Brown, 2013; Frewer, 2004; Frewer et al., 2003; Landström et al., 2015).

### Conveying Unsettled Science as Contentious

Another frame sometimes adopted in public-facing communications about socioscientific issues is of science as contentious and riven by disagreement (e.g., Boykoff, 2011; Zehr, 2000). This frame is not generally adopted in communications that either come from scientists themselves or that emphasize arguments and claims made by scientists (Rice et al., 2018).

In a study examining portrayal of climate change in print news, Rice and colleagues (2018) identified three types of what they call “opinion divergence” expressed in news articles. These included disagreements between individuals, controversies between groups, and skepticism expressed as prolonged opposition to an argument. These three “opinion divergence” frames do not usually convey specific claims about scientific uncertainty. For example, they may instead convey disagreements about policies that should be adopted in response to a socioscientific issue.

However, often—intentionally or not—these portrayals do convey information that audiences read as addressing inconclusiveness of scientific knowledge (Peters & Dunwoody, 2016). Rice and colleagues (2018) provide several examples including from a 2012 *New York Times* article portraying opinion divergence as controversy: “The new research is an attempt to resolve a scientific controversy that erupted several years ago about exactly how fast West Antarctica is warming.” And a portrayal of opinion divergence as skepticism from a 2010 *New York Times* article noting: “some senators challenging the notion that the earth is warming.” While neither of these examples conveys details of scientific claims about uncertainty, they both imply that uncertainty concerning warming exists.

We suggest that frames of disagreement, controversy, and skepticism that stop at merely describing dissension do not provide public audiences with information concerning scientific claims of uncertainty that could productively be used to inform participation in relevant debates and discussions. For example, in the controversy within the scientific community about warming in West Antarctica, a description of alternative hypotheses and the additional data and analyses that are being collected could help readers understand that both sides agree about how this disagreement could be resolved through further scientific investigation. In contrast, it is not at all clear what new information might lead the senators to change their minds about global warming. Thus, the Antarctic ice controversy promotes information seeking in the scientific community, while the senators’ opposition to global warming seems to be based on epistemic hubris. The controversy frame does not make this distinction clear.

### Conveying Unsettled Science as Including Scientific Claims About Uncertainty

A third approach to conveying scientific uncertainty attempts to describe analyses of risk and uncertainty with more detail, while still aiming for accessibility to a public audience (e.g., Budescu et al., 2012; Dieckmann et al., 2012; Fischhoff & Davis, 2014; Flemming et al., 2015; Markon & Lemyre, 2013; Rice et al., 2018). Reports and articles produced by bodies such as the Intergovernmental Panel on Climate Change are examples of this type of communication (e.g., Juanchich et al., 2020; McMahon et al., 2015). We are particularly interested in how people can make sense of thoughtfully conveyed scientific claims about uncertainty and use their understanding to make informed decisions that consider scientific uncertainty.

There is a growing body of research concerning more and less effective ways to convey claims about scientific uncertainty to the public (e.g., Corbett & Durfee, 2004; Dieckmann et al., 2017; McMahon et al., 2015; Moss et al., 2008; Spiegelhalter et al., 2011). These overlap with the literature concerning public perspectives of uncertainty discussed in the previous section. Much of this literature has a communications focus rather than an education focus. That is, it is concerned with how best to convey claims about scientific uncertainty given public capacity to make sense of these claims.

**Fig. 2:**
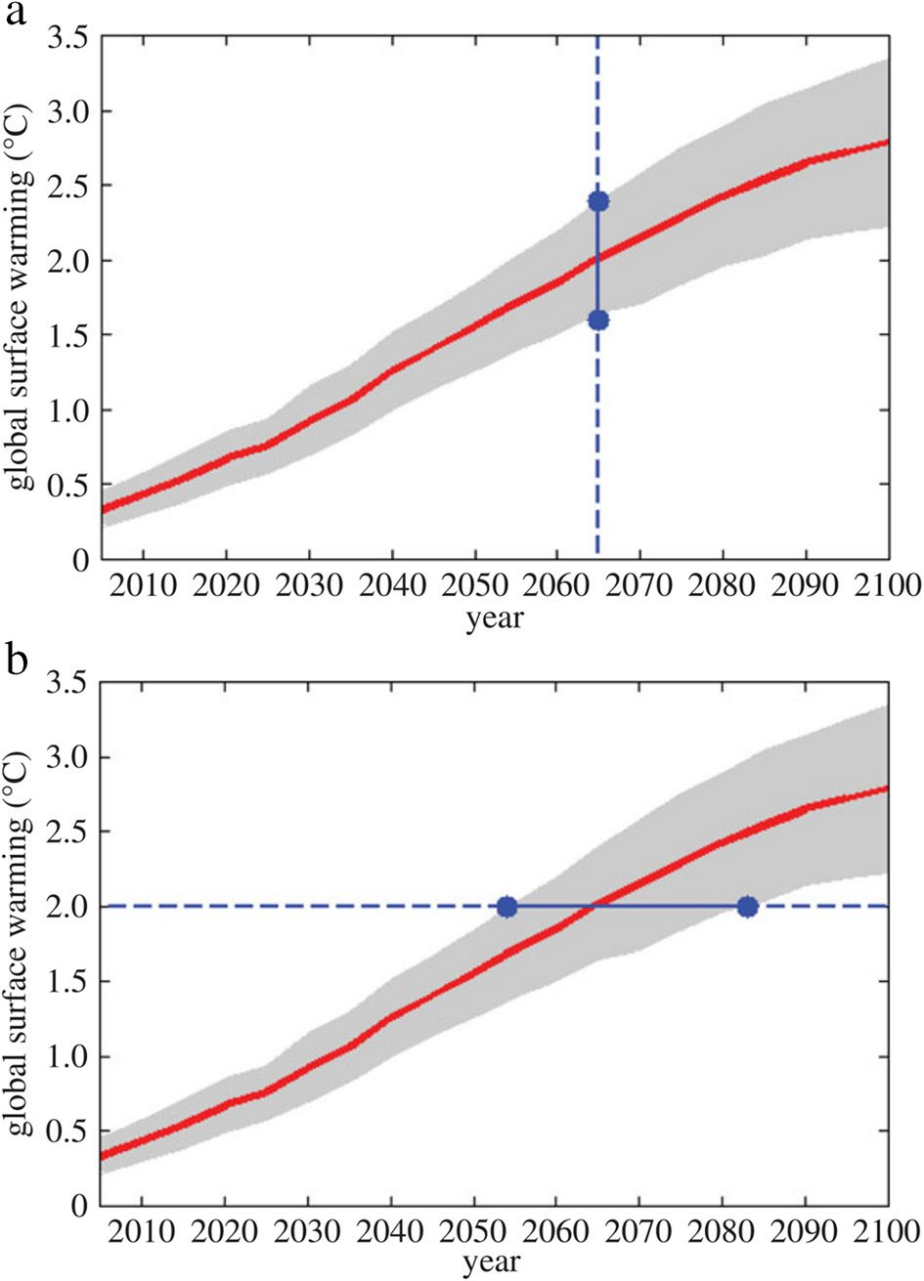
Outcome-uncertain (a) and time-uncertain (b) representations of scientific uncertainty (Ballard & Lewandowsky, 2015)

For example, some research concerning how best to communicate scientific uncertainty examines how people make sense of different representations of uncertainty and judges which representations are most effective at conveying an intended message (Budescu et al, 2009; Corbett & Durfee, 2004). In one climate-related study, Ballard and Lewandowsky (2015) presented people with projections for increases in temperature and sea levels that either emphasized what the level of uncertainty for the outcome would be at a particular time or else emphasized uncertainty in the arrival time at which the outcome would occur (Fig. 2). They found that people perceived the threat as more serious and were more likely to advocate for mitigative action in the time-uncertain versus the outcome-uncertain condition.

Other studies emphasize that different ways of representing scientific uncertainty are more or less effective depending on characteristics of the audience (e.g., Broomell & Kane, 2017; European Food Safety Authority et al., 2019). For example, in a study of the interaction between beliefs about science and communicated level of uncertainty, Rabinovich and Morton (2012) found that messages communicating high uncertainty were more persuasive for people who view science as a set of unanswered questions open to debate compared with people who view science as an endeavor to uncover objective truth. This type of study is reflective of approaches that are common in marketing, wherein communicators undertake market segmentation to direct persuasive messages to different audiences based on characteristics of those audiences (Goyat, 2011).

Members of the scientific community have also offered strategies for decision-making amidst uncertainty. For example, the *America’s Climate Choices* report provides an “iterative risk management approach for addressing climate change” (National Research Council, 2011, p. 45). Notable aspects of this approach include that it attempts to integrate multiple domains (e.g., science, economics, policy, equity) and that it assumes decision-making will be iterative in nature—revisiting and revising decisions as both qualitative and quantitative facets of uncertainty are reduced over time. Another example of an approach to decision-making amidst uncertainty is risk management, which involves identification, evaluation, selection, implementation, and monitoring of responses to risk (Aven & Renn, 2010).

Communications research concerning how best to convey claims about scientific uncertainty to the public rarely addresses the possibility that the public could develop or improve its capacity to make sense of scientific claims about uncertainty. This leaves an important role for science education—it is our job to support students in developing science literacy. We argue that science literacy for non-scientists should include the capacity to evaluate scientific analyses of uncertainty that they encounter (usually in the media) and to use these analyses as they engage in science practices, including to inform personal (e.g., consumer, health) and societal (e.g., voting, participating in public debates) actions and decisions (Feinstein, 2011; National Academies of Sciences, Engineering, and Medicine, 2016). Science education has a responsibility to prepare people to use science knowledge and practices to participate in discussions and debates about socioscientific issues, rather than a responsibility to advocate for specific policy-related behaviors or positions (Kolstø, 2001a; National Research Council, 2012; Sadler et al., 2007).

## Educating Students to Create and Use Scientific Analyses of Uncertainty

In Sect. 5, we discuss promising strategies for helping K-12 students to create and analyze claims about uncertainty, both in their own investigations and in media reports and articles, as they engage in science practices. We begin by reviewing literature on students’ capacities to understand and use scientific claims about uncertainty. We then discuss both how classroom discourse can leverage uncertainty as an epistemic emotion (Carruthers, 2017) that prompts curiosity and information seeking, and how teachers and researchers have scaffolded students’ engagement in science practices such as investigations, arguments from evidence, and explanations to include analyses of uncertainty (National Research Council, 2012; NGSS Lead States, 2013). Finally, we discuss promising strategies for scaffolding students to interpret and use accounts of scientific uncertainty in media reports.

### Educational Studies of Students’ Understanding of Uncertainty

Compared with the literature base in psychology, there are fewer educational studies of how students make sense of and learn to make sense of scientific claims about uncertainty (e.g., Manz, 2015, 2018; Metz, 2004; Pallant et al., 2020; Schroeder et al., 2019). Studies that have been conducted, however, suggest that even young students can demonstrate significant capacity to make sense of uncertainty in ways that reflect scientific approaches—and that their understandings and learnings span both qualitative and quantitative aspects of scientific uncertainty. For example, in a study of second, fourth, and fifth graders’ conceptualizations of uncertainty in investigations they had designed and implemented themselves, Metz (2004) found that 71% of second graders and 87% of fourth and fifth graders conceptualized one or more spheres of uncertainty including (a) production of a desired outcome as uncertain, (b) data as uncertain, (c) trends in data as uncertain, (d) generalizability of a trend as uncertain, and (e) which theory best accounts for a trend as uncertain. Further, “[a]mong those who had conceptualized one or more spheres of uncertainty, 80% of second graders and 97% of fourth-fifth graders posited a strategy to modify their study to address uncertainty” (Metz, 2004, p. 219).

More recently, Schroeder and colleagues (2019) conducted a study examining fifth and ninth grade students’ views of scientific uncertainty. Like Metz, Schroeder and colleagues examined the students’ conceptualizations of uncertainty in investigations they conducted themselves. Similar to Metz, they found that students were able to conceptualize multiple spheres of uncertainty. In addition, Schroeder’s study also examined students’ ideas about uncertainty in the context of perceptions of the work of professional scientists. In this domain, Schroeder found that many fifth graders thought that there should be just one correct conclusion in professional science and that any differences in conclusions must have stemmed from procedural errors. In contrast, more ninth graders thought that the scientists’ perspectives could lead to different views. Very few students in either grade suggested the importance of weighing evidence when comparing different conclusions. The study did not examine whether students could identify continuing uncertainty that extends beyond individual investigations. Studies like those of Metz (2004) and Schroeder and colleagues (2019) provide evidence that students, even young students, can conceptualize both qualitative and quantitative facets of scientific uncertainty.

We found similar evidence of capacity to make sense of scientific uncertainty in a study we conducted of high school students who used multiple types of models (physical, conceptual, computational) to explain, predict, and develop mitigation plans for a case of groundwater contamination at a Superfund site in their state (Covitt et al., 2020). Pre-/post-assessment items in the Comp Hydro project asked students to conceptualize aspects of scientific uncertainty including uncertainty associated with judging the accuracy of a computer model. On the posttest, 46% of students were able to explain that scientists judge the accuracy of computer models through strategies such as calibration or comparing model results to results observed in the real world. For example, one student’s response stated that to judge the accuracy of a computer model, scientists, “can test it and go back to the actual site and take more tests and do experiments to make sure that it is right. And if not they will calibrate it and keep fixing it until it is accurate.” A further 36% of responses indicated that scientists judge model accuracy through less specific methods such as testing their models or inputting more or more accurate data into their models. Relevant example responses included “Scientists can test other models to see how accurate the computer was,” “Test it multiple times,” and “by taking data in the feald [sic].”

On another Comp Hydro post-assessment question asking students to identify problems with using computer models to understand hydrologic system problems, 19% of students noted the problems that models may not account for uncontrolled variables or that models may be difficult to calibrate. One student wrote, “Computer models are unable to have every possible variable that a real water problem would have.” A further 43% offered less specific but reasonable problems such as issues with model codes. An example of this type of response was “its [sic] hard to use a computer model in some real world problems because its hard to set the code and show the problem correctly.”

**Fig. 3:**
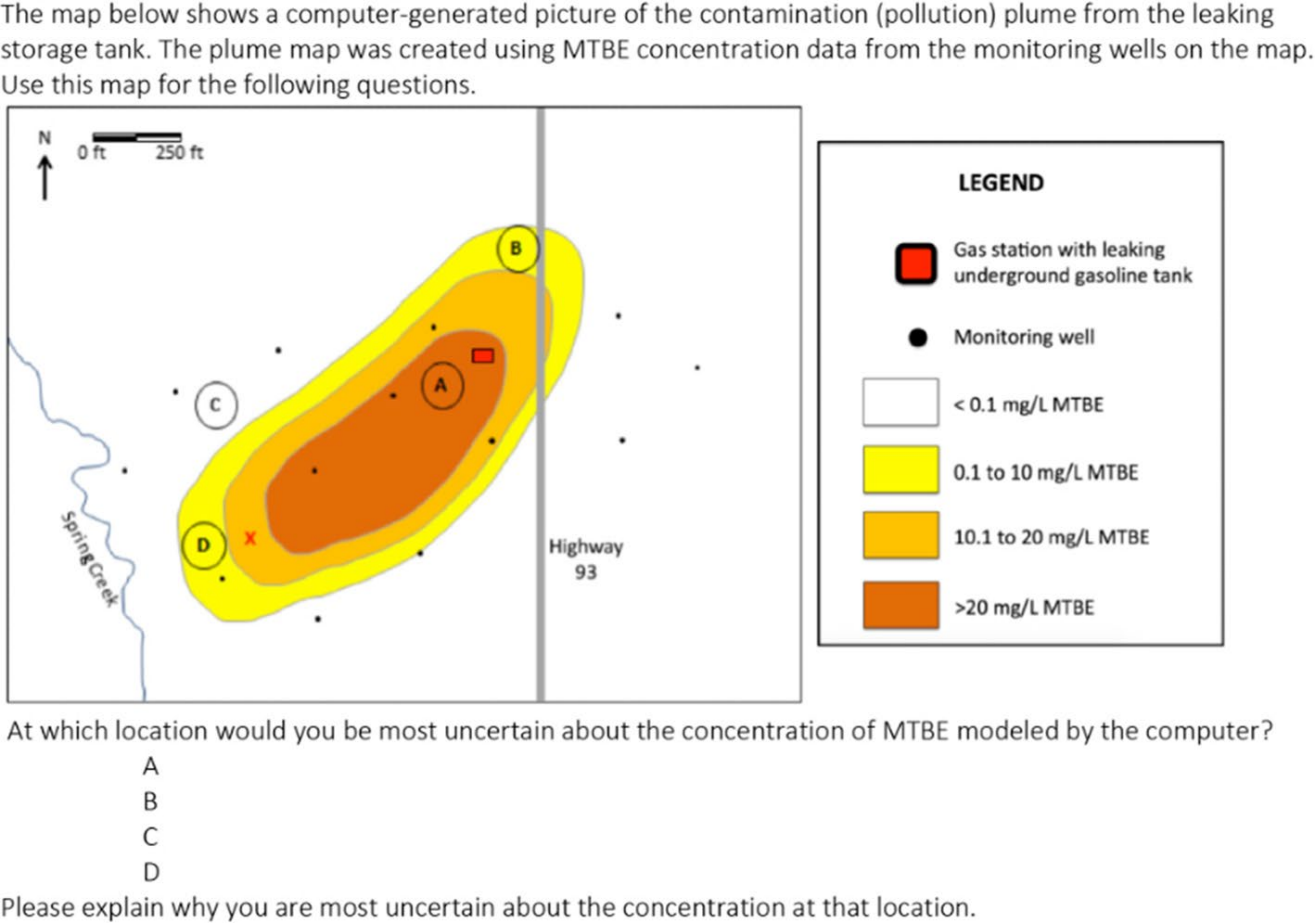
Assessment item asking students to judge uncertainty

While the studies described above suggest that K-12 students can develop sophisticated understanding of scientific claims about uncertainty, we also know that this is not an easy or straightforward area of science to teach. For example, in our study of students investigating a case of groundwater contamination, after engaging in a lesson demonstrating how uncertainty can be reduced through additional data collection and analysis (lesson described in Sect. 5.3), we asked students to analyze a contour map of a contamination plume and identify at which location they thought the estimated contamination level shown would have the most uncertainty associated with it (Fig. 3). On the posttest, only 18% of students indicated that there would be higher uncertainty associated with the location that had no nearby monitoring wells (i.e., identifying uncertainty due to insufficient data). One student responding in this way wrote, “there aren’t very many wells around B so it would be hard to know exactly what the concentration would be.” A further 26% of students provided responses suggesting they were starting to develop scientific ideas and approaches to judging uncertainty (e.g., noting imprecision in the range of values on the contour map). A student reflecting this type of reasoning responded they were most uncertain about location “A [because] it could be any amount more than 20.”

While deciphering scientific uncertainty is challenging, we would suggest that our study and studies like those conducted by Metz (2004) and Schroeder and colleagues (2019) demonstrate that K-12 students have significant intellectual potential to make sense of sources of scientific uncertainty and scientific claims about uncertainty. Considering studies like these in conjunction with our knowledge of the current state of K-12 science education raises an important question for science education. We wonder what public understanding of scientific uncertainty could be achieved if students’ K-12 science education experiences routinely (or at least more frequently) scaffolded their capacities to analyze and communicate about uncertainty, incorporating claims about uncertainty into their arguments and explanations?

### Engaging Students in Investigations that Recognize and Seek to Resolve Uncertainty

The studies cited above by Metz and Schroeder and colleagues come from a body of design-based research that leverages uncertainty to play a key role in classroom investigations. Much of this work focuses on affective dimensions of uncertainty, showing how uncertainty can motivate students and adults to engage in information seeking (e.g., Huang & Yang, 2020), or to improve the quality of their science practices, including investigations, arguments from evidence, and explanations. For example, Radoff and colleagues (2019) describe how a student in a college physics course progressed from anxiety, to comfort, to excitement about feeling uncertain, coming to recognize uncertainty as an opportunity for learning. Similarly, Watkins et al. (2018) analyzed how expressions of uncertainty prompted and sustained productive discussions in elementary and college classrooms.

Manz and colleagues have examined how students and teachers leverage uncertainty in classroom investigations and arguments from evidence (e.g., Manz, 2015, 2018). Manz and Suárez (2018, p. 771) suggest that “an essential aspect of the teachers’ work was developing a more nuanced view of scientific uncertainty.” They identified “three strategies that appeared to help teachers negotiate and develop this more nuanced view: beginning with complex phenomena, iterating on investigations, and leveraging variability in students’ ways of conducting investigations.” Similarly, Tekkumru-Kisa and colleagues (2021) identify ambiguity as an essential property of high-quality science tasks.

While we recognize and applaud the quality of the instruction described in these studies and endorse their approaches to including and leveraging scientific uncertainty, we also note a limitation to this body of work. Most of these studies focus on raising and reducing or resolving qualitative uncertainty within an instructional sequence of students’ classroom science work and investigations. There are fewer examples of approaches that enable students to either analyze and quantify continuing uncertainty after the conclusion of their scientific investigations or to judge uncertainty (including quantitative uncertainty) in reports of socioscientific issues. We next describe some studies that address these issues.

### Engaging Students in Analyzing and Quantifying Uncertainty

Some pertinent scholarship on analyzing and quantifying uncertainty comes from researchers working on the borders between science and mathematics education, focusing on data and data modeling. We discuss three examples below.

*Modeling signal and noise in measurement.* Lehrer, Schauble, and colleagues report on a series of studies in which upper elementary students examined distributions of measurements and developed approaches to modeling measurement error (Lehrer & Kim, 2009; Lehrer & Schauble, 2002; Lehrer et al., 2007, 2011). For example, Lehrer and colleagues (2011) describe a design experiment in which individual students used different tools (a meter stick and a 15-cm ruler) to measure the span of their teacher’s outstretched arms, recording a variety of measurements. The students then worked to describe two characteristics of the distribution of measurements:

The first was a measure of the “best guess of the real measurement.” This approach invited students to consider a statistic as a measure of the signal of the batch of measurement outcomes, here the true length of the teacher’s arm-span (Konold & Pollatsek, 2002; Petrosino et al., 2003). The second challenge was to design a measure of the “precision” of the batch of measurements, so that students were positioned to develop an indicator of variability. (Lehrer et al., 2011, p. 726)

Note the central role that uncertainty played in both challenges. The first challenge required the students to design strategies for reducing uncertainty: If each individual measurement is uncertain, how can they use the distribution of measurements to produce a less uncertain estimate of the teacher’s arm span? The second challenge required students to analyze and quantify continuing uncertainty: How can they describe the variation in their measurements in quantitative terms? During the design experiment, the students invented, critiqued, and modified statistics that are clearly related to the concepts of median, range, and distribution as reported in scientific journals.

*Reaching conclusions based on noisy data.* In another set of studies, Cobb and McClain engaged middle school students in developing ways to represent variation and covariation in data and in developing arguments about warranted conclusions based on that variation (Cobb et al., 2003; McClain, 1999; McClain et al., 2000). They reported on two design experiments; both involved looking for patterns in “noisy” data related to socioscientific issues. The first involved deciding which of two drugs for treating AIDS was “better,” given data about T-cell counts for two treatment groups of different sizes, both showing a large range of measurements. The second involved characterizing the relationship between date and atmospheric CO_2_ concentrations given multiple measurements at different times over a 40-year period. In both cases, students first had to develop statistics for central tendency and variability (as in the studies by Lehrer et al. described above). Then, they needed to use those measures to reach two kinds of conclusions. The first had to do with reaching consensus on the best answers to the questions posed (developing strategies for resolving uncertainty). Second, they had to decide how confident they were in their conclusions (analyzing continuing uncertainty).

**Fig. 4:**
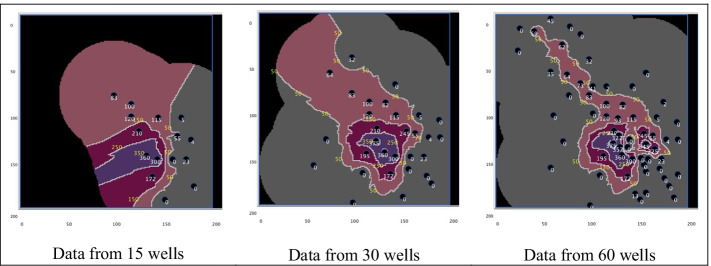
NetLogo contour map model outputs showing contoured contamination plume

*Characterizing uncertainty based on differences in sampling.* High school students in the Comp Hydro project adopted the role of hydrogeologists investigating and recommending a remediation response to a case of groundwater contamination. Lessons provided opportunities to analyze scientific uncertainty in the context of a socioscientific issue requiring action. In one activity, students used selenium concentration data that had been collected from 15 wells at a Superfund site to create contamination plume contour maps by hand. They used linear interpolation and extrapolation to estimate where contour lines should be drawn and to explore data limitations. In the following activity, they used a NetLogo contour map computational model to generate plume contour maps with data from 15, 30, and 60 wells (See Fig. 4).

These activities were designed to engage students in grappling with scientific uncertainty in data. They also modeled how scientists investigating a case of contamination reduce uncertainty through collecting data sufficient to identify a contaminant plume’s boundaries and to have adequate resolution for identifying distinct contamination sources at a site. The Comp Hydro unit culminated with student teams making recommendations for responding to the groundwater contamination; teams’ recommendation plans were constrained by a budget limitation commensurate with that of the actual cleanup and required the students to account for the perspectives of various stakeholders. This experience engaged students both in grappling with quantitative aspects of scientific uncertainty and with considering scientific claims about uncertainty within the larger context of a complex and multi-dimensional socioscientific problem that could not be solved with science alone.

### Engaging Students with Claims About Uncertainty in Media Reports and Articles

The studies cited above focus on fostering students’ personal and collective engagement with phenomena. This focus appropriately shifts K-12 science education from the rhetoric of conclusions to engaging students with uncertainty as they participate in science practices such as analyzing data and models. However, it is also essential to build students’ capacity to make sense of others’ reports of science associated with socioscientific issues—what Magnusson and Palincsar (2001) call “second-hand inquiry.”

There are notable examples of science education aimed at preparing students to make sense of socioscientific issues through experiences that go beyond personal inquiry. Prominent in this area is Feinstein and colleague’s (2013) exhortation for science education to create “competent outsiders” in science who have the capacity to judge scientific claims. Two other veins in this domain include Sadler, Zeidler, and others’ work on socioscientific issues instruction (e.g., Kolstø, 2001a, 2001b; Sadler, 2009; Zeidler et al., 2009); as well as citizen science education that engages students in undertaking collaborative science to address real-world problems (Bonney et al., 2014; Phillips et al., 2019).

There is also a growing body of work concerning media literacy education within both science education and other disciplines of education (e.g., Cooper, 2011; Feinstein & Waddington, 2020; Höttecke & Allchin, 2020; Sinatra & Lombardi, 2020; Wineburg & McGrew, 2019). Scholars in this area study ways to support students in judging the credibility and plausibility of arguments made in media articles and reports. Focus is placed on helping students find and evaluate trustworthy media sources, including making judgments about credibility, trustworthiness, and plausibility (Sinatra & Lombardi, 2020; Wineburg & McGrew, 2019). A related domain of judgment involves distinguishing between scientific and non-scientific claims (Covitt et al., 2013; Zeidler & Kahn, 2014).

Evidence-based scaffolds for evaluating media reports of science have been forwarded. One example is lateral reasoning, which involves checking other sources to judge the credibility of an initial source (Stanford History Education Group, 2021; Wineburg & McGrew, 2019). Another scaffold involves evaluating “connections between multiple lines of scientific evidence and alternative explanatory models about an observed phenomenon” to judge plausibility (Sinatra & Lombardi, 2020). Similarly, the National Association for Media Literacy Education (2021) provides a rubric with key questions for analyzing media. These scaffolds highlight essential elements of media literacy.

However, our experience has been that students who encounter legitimate media sources often struggle to make sense of what they see and read, including scientific analyses of uncertainty. While our review was not exhaustive, across the work we have read, we found few examples of instruction that explicitly engages students in judging scientific claims about uncertainty in media reports and articles. Given this dearth in identified literature, we offer several ideas for helping students learn to judge claims about scientific uncertainty in science reports and media.

*Engaging with media reports of quantified uncertainty.* Regarding quantitative uncertainty, we again borrow from the Comp Hydro project in which students studied groundwater contamination. In the unit, we asked students to take on the role of hydrogeologists who needed to investigate and respond to the contamination. However, they did not take on this role in a vacuum. The unit interwove opportunities for students to investigate the contamination themselves through using data that had been collected by scientists and technicians (e.g., selenium concentrations in samples that had been collected at monitoring wells on the site) with opportunities for students to compare their own investigations with those conducted by the scientists who investigated the site.

Examples of interwoven activities included students using less complex NetLogo computer models to examine how contaminants flow through groundwater systems followed by watching a video of the Superfund site modeler discussing how the MODFLOW computational model (Harbaugh et al., 2000) of the site was set up, implemented, and calibrated (i.e., with targets for how similar observed and modeled results needed to be) to characterize contamination at the site. The students also worked in teams to develop remediation plans and then compared their plans with the actual Superfund site plan that explained how contamination at the site was remediated amidst continuing uncertainty.

These activities provided opportunities for students to connect their own investigations with the activities and reports produced by the scientists working on the Superfund site. Combining students’ personal investigations using second-hand data from the site with consideration of reports from the scientists at the site provided the high school students with a window into how scientists manage, analyze, and seek to reduce scientific uncertainty. After participating in the Comp Hydro unit, students demonstrated significant pre-/post-improvements in their capacities to make sense of data and representations characteristic of those found in scientific reports and articles concerning groundwater contamination, and in their understanding of how scientists use imperfect (i.e., characterized by uncertainty) data and computer models to explain and predict contamination events in groundwater systems (Covitt et al., 2020).

Another strategy we have envisioned, but not implemented, could engage students in exploring and comparing multiple ways that uncertainty is visualized in reports of scientific research (e.g., ranges, multiple outcomes, simulations, obscurity) (MacEachren et al., 2005; Spiegelhalter et al., 2011; Yau, 2017). This type of exploration and comparison, with accompanying scaffolding for classroom discourse, could help prepare students to more effectively decipher representations of uncertainty that they encounter in articles and reports about socioscientific issues. Given that students rarely engage in these types of activities in school science, the findings from the literature that we previously described of problematic public understanding of uncertainty seem unsurprising (Broomell & Kane, 2017; Kahneman, 2011; Rabinovich & Morton, 2012). Much work remains to be done to explore what kinds of strategies could effectively prepare students to make sense of the scientific analyses of quantitative uncertainty that they will encounter after they finish their formal science education.

*Engaging with media reports of qualitative/conceptual uncertainty.* With regard to judging media reports of qualitative and conceptual uncertainty, scaffolds developed in the domain of science media literacy hold promise. For example, Goldman and colleagues (2019) report on a large-scale study of an intervention designed to promote ninth-grade science students’ use of text-based investigations. The core constructs for the intervention include evaluating the strength of evidence supporting claims in media accounts and limitations in the models and theories used to interpret that evidence—prompting students to consider strategies for resolving uncertainty. We have shared scaffolds developed through similar work we conducted with secondary students around evaluating scientific arguments in media communications (Covitt et al., 2013).

Another of our curriculum projects, Carbon TIME, includes a Questions, Connections, Questions reading strategy, which prompts students to (a) ask questions about things that they find puzzling in a text or other media account, (b) make connections between the account and other texts or their personal experiences, and (c) ask new questions based on the account (Carbon TIME, 2021). This strategy prompts students both to identify areas of uncertainty that could be resolved by the account (the initial questions) and identify continuing uncertainty (the final questions).

*Designing tools to scaffold students’ engagement with uncertainty in media reports.* In our review of these various supports, one facet that seems to be missing is scaffolds that help readers focus on and evaluate explicit claims about current and continuing uncertainty in media communications. Evaluation tools designed for this purpose could be integrated with other media literacy supports. The basis for design could rest in and support students’ growing familiarity with sources of scientific uncertainty highlighted in Fig. 1. For example, a tool could prompt students to distinguish between issues of trustworthiness and issues of scientific uncertainty. Similarly, it might prompt evaluation of how qualitative uncertainty is conveyed in an article (i.e., as an unresolved question about alternative hypotheses shared among scientists or as a disagreement between scientists and another group such as climate deniers). Such a tool could scaffold readers to carefully evaluate the treatment of scientific claims about uncertainty in media communications—with attention to making sense of how quantitative uncertainty (including continuing uncertainty) is expressed and how well the scientists’ argument holds up given the types and levels of uncertainty that exist.

Finally, a scientific uncertainty tool could support readers in considering facets of uncertainty important for making sense of socioscientific issues (i.e., through considering how types and extent of scientific uncertainty conveyed in an article may intersect with other concerns to inform one’s opinion or decision-making process). This is similar to a scaffold for assessing costs and benefits in light of uncertain scientific predictions that is outlined in the *America’s Climate Choices* iterative risk management strategy (National Research Council, 2011). While the goal of designing effective approaches for teaching media literacy with respect to scientific uncertainty poses a significant challenge, we argue that achieving this goal is essential for preparing students to use science in their lives after school.

## Conclusion

We began this article with a question: *How should we trust science?* The answer we developed focuses on a key characteristic of scientific conclusions and predictions: Communication among scientists emphasizes analyses of uncertainty as well as claims about the natural world. Scientific journal articles incorporate representations and quantifications of uncertainty. Studies of science in cultural and historical contexts document many ways in which specialists’ analyses of uncertainty can be incomplete, sometimes in ways that have profound implications for social justice or environmental sustainability. Thus, uncertainty plays a central role in scientific discourse—both uncertainty recognized and resolved and continuing uncertainty still to be addressed.

Scientific reports in the media often focus more on conclusions and predictions and less on uncertainty; evidence about public understanding of science indicates that this is probably necessary in some form. Today, most members of the public are insufficiently prepared to understand the analyses and quantifications of uncertainty in the primary literature. The consequences, though, are manifest in public skepticism about science and decision-making about socioscientific issues.

The perspectives and approaches we have discussed can provide guidance concerning the question of *How should we trust science?* for science education. By supporting students in figuring out *how* rather than *why* they should trust science, science education can focus its instructional aims on knowledge and practices necessary for people to use science productively in their lives. Those who are prepared to use science to inform their thinking and deciding understand that the response to “*how we should trust science*” should involve curiosity and information seeking rather than epistemic hubris.

Scientifically literate individuals recognize that science is limited, uncertain, *and* useful. This means that not all questions (and particularly not policy-related questions) can be answered with science alone, and, also, that some questions cannot be answered with science at all. Thus, science has an important but limited role to play in societal decision-making. While many scholars of science education in recent decades have focused on the fact that science is limited, there has, perhaps, been less emphasis on the idea that science is simultaneously uniquely useful. Science provides powerful tools for explaining and predicting events in the material world to inform our responses to challenges like climate change and global pandemics.

Literate consumers of scientific communication understand that it is important to seek new information when they encounter uncertainty, and to evaluate scientific conclusions and predictions through multiple lenses including with regard to (un)trustworthiness and qualitative and quantitative scientific uncertainty. Untrustworthy science can and should be discounted and challenged, but uncertainty in science is unavoidable, and scientific characterization of uncertainty can be extremely useful when decision-makers have strategies for making decisions amidst that uncertainty.

While (un)trustworthiness and uncertainty in science can be distinguished from each other, they are also related. Thus, we believe that effective approaches to developing science literacy will require instructional attention to both of these concerns—and will also require engaging students in experiences with making sense of these concerns both separately and together. Current design work in science education includes a worthwhile emphasis on helping students to recognize and leverage uncertainty as they engage in science practices with their own data and models. We see this as important, but not sufficient. Students also need to develop proficiency in seeking out and understanding analyses of continuing uncertainty in media accounts of scientific conclusions and predictions.

In this article, we have mostly addressed scholarship and instructional approaches related to scientific claims about uncertainty because we see these as an essential yet rarely treated domain in K-12 science education. There are promising efforts underway to address issues encompassing both trustworthiness and uncertainty in science education; we hope that this article will help readers to appreciate their distinctions and their importance.

## Data Availability

Data and materials are either available online or by request from the authors.
